# Standard rodent diets differentially impact alcohol consumption, preference, and gut microbiome diversity

**DOI:** 10.3389/fnins.2024.1383181

**Published:** 2024-05-13

**Authors:** Aline Zaparte, Evan Dore, Selby White, Franciely Paliarin, Cameron Gabriel, Katherine Copenhaver, Samhita Basavanhalli, Emily Garcia, Rishith Vaddavalli, Meng Luo, Christopher M. Taylor, David Allen Welsh, Rajani Maiya

**Affiliations:** ^1^Department of Microbiology, Immunology, and Parasitology, Louisiana State University Health Sciences Center New Orleans, New Orleans, LA, United States; ^2^Department of Physiology, Louisiana State University Health Sciences Center New Orleans, New Orleans, LA, United States

**Keywords:** intermittent access, Lab Diet 5053, Lab Diet 5001, Teklad 2019S, quinine resistance

## Abstract

Alcohol use disorder (AUD) is a complex and widespread disease with limited pharmacotherapies. Preclinical animal models of AUD use a variety of voluntary alcohol consumption procedures to recapitulate different phases of AUD, including binge alcohol consumption and dependence. However, voluntary alcohol consumption in mice is widely variable, making it difficult to reproduce results across labs. Accumulating evidence indicates that different brands of commercially available rodent chow can profoundly influence alcohol intake. In this study, we investigated the effects of three commercially available and widely used rodent diet formulations on alcohol consumption and preference in C57BL/6 J mice using the 24 h intermittent access procedure. The three brands of chow tested were LabDiet 5,001 (LD5001), LabDiet 5,053 (LD5053), and Teklad 2019S (TL2019S) from two companies (Research Diets and Envigo, respectively). Mice fed LD5001 and LD5053 displayed higher levels of alcohol consumption and preference compared to mice fed TL2019S. We also found that alcohol consumption and preference could be rapidly switched by changing the diet 48 h prior to alcohol administration. Sucrose, saccharin, and quinine preferences were not altered, suggesting that the diets did not alter sweet and bitter taste perception. We also found that mice fed LD5001 displayed increased quinine-resistant alcohol intake compared to mice fed TL2019S, suggesting that diets could influence the development of compulsive behaviors such as alcohol consumption. We profiled the gut microbiome of water- and alcohol-drinking mice that were maintained on different diets and found significant differences in bacterial alpha- and beta-diversities, which could impact the gut–brain axis signaling and alcohol consumption.

## Introduction

Alcohol use disorder (AUD) is a persistent and recurring brain disorder marked by a diminished capacity to cease or regulate alcohol consumption, even in the face of negative repercussions (Koob and Volkow, 2016). Alcohol is one of the most misused drugs worldwide, and within the United States, AUD has a lifetime prevalence rate of nearly 8.6% (Glantz et al., 2020). Alcohol consumption significantly impacts both the central and peripheral nervous systems, and its toxic effects can promote cell and tissue injuries throughout the body (Dubinkina et al., 2017). Currently, there are only three FDA-approved treatments for AUD that all suffer from variable efficacy (Lohoff, 2022). Hence, there is an urgent need for the discovery of novel therapeutics to treat AUD.

Animal models of AUD enable us to identify neuroadaptations that underlie the development of alcohol dependence, thereby enabling the discovery of novel therapeutic targets to mitigate AUD. Preclinical studies rely on a suite of voluntary alcohol consumption procedures to capture different phases of AUD, such as binge alcohol consumption, alcohol dependence, and relapse and reinstatement to alcohol seeking (Tabakoff and Hoffman, 2000; Rhodes et al., 2005; Vengeliene et al., 2014). However, the amount of alcohol consumed in voluntary alcohol consumption procedures often varies widely between laboratories, making it difficult to reproduce key findings. A variety of factors could contribute to this variability, including housing conditions such as type of bedding, temperature, and humidity in the vivarium (Crabbe and Wahlsten, 2003). However, accumulating literature suggests (Marshall et al., 2015; Quadir et al., 2020; Maphis et al., 2022) that the type of rodent chow could be a significant contributor to variations in alcohol consumption. Rodent chow is not standardized across laboratories and can vary significantly in composition and texture. Diet can profoundly influence behavioral outcomes through a variety of pathways, including signaling through the gut–brain axis (Leclercq et al., 2020) and altering taste perception (Tordoff et al., 2002). Previous studies have examined the effects of various commercial rodent diets on alcohol consumption and preference (Marshall et al., 2015; Quadir et al., 2020; Maphis et al., 2022). These studies have found that the type of rodent chow used can significantly affect not only the amount but also the pattern of alcohol intake in voluntary drinking procedures. However, the mechanism(s) by which diet can influence alcohol consumption has not been examined in these studies.

One potential mechanism by which rodent diet could influence alcohol consumption is through modifications to the gut microbial composition. Diet is the main modulator of gut microbiome composition and consequently can alter the gut–brain axis signaling (Lynch and Pedersen, 2016), which may play a role in accelerating the addiction cycle (Bravo et al., 2011; García-Cabrerizo et al., 2021). Alcohol consumption by itself can also alter gut microbial communities, leading to dysbiosis and gut leak and triggering end-organ chronic inflammation (Stärkel et al., 2018). Additionally, the modifications of the gut microbiota due to alcohol consumption play a role in the development of alcohol-associated diseases, such as non-alcoholic fatty liver disease, cardiovascular diseases, and neuropsychiatric disorders (Chang and Kao, 2019). Furthermore, diet plays a pivotal role in shaping the gut microbiome (Lynch and Pedersen, 2016), while limited information is known about how food composition and nutritional habits regulate the motivation to seek drugs. Given the influence of the diet on the gut microbiome and the connection between the gut microbiota and behavior, we aimed to investigate the impact of commercially available rodent diets on alcohol consumption and preference and gut microbiome diversity.

## Materials and methods

### Animals

For this study, 7- to 8-week-old male and female C57BL/6 J mice were purchased from The Jackson Laboratory (Bar Harbor, ME). Mice were provided access to food and water *ad libitum* and individually housed. Mice were allowed to habituate to a reverse light/dark cycle (on at 10:00 a.m., off at 10:00 p.m.) for 1 week prior to the initiation of the experiment. During the habituation period, mice were individually housed in double grommet cages equipped with two bottles provided with sipper tubes containing water. Animals were weighed once per week.

### Rodent diets

Animals were provided *ad libitum* access to specified diets of either LabDiet 5001, LabDiet 5053 (Research Diets, New Brunswick, NJ), or Teklad 2019S (Envigo, Indianapolis, IN). The diets differed significantly in macro- and micronutrient composition, some of which are listed in Table 1.

**Table 1:** Major similarities and differences in macro- and micronutrient compositions of the three standard rodent diets used in the study.

	Diet		
Nutrients	TL2019S	LD5053	LD5001
*Energy*			
Calories from carbohydrates (%)	55.00	62.38	58.00
Calories from fat (%)	22.00	13.12	13.50
Calories from protein (%)	23.00	24.50	28.51
Metabolizable energy (kcal/g)	3.30	3.02	3.02
*Fatty acids*			
C18:2ω6 linoleic (%)	3.90	2.32	1.22
Fat (acid hydrolysis) (%)	9.00	6.30	5.70
Fat (ether extract) (%)	10.00	5.00	5.00
Fiber (crude) (%)	2.60	4.40	5.10
Total monounsaturated (%)	1.70	1.00	1.60
Total saturated (%)	1.20	0.77	1.56
*Minerals*			
Copper (mg/kg)	15.00	13.00	0.13
Iodine (mg/kg)	6.00	0.97	1.00
Manganese (mg/kg)	80.00	82.00	0.70
Potassium (%)	0.40	1.07	1.18
*Vitamins*			
Biotin (ppm)	0.90	0.30	0.30
Choline chloride (ppm)	1200.00	1575.00	2250.00
Folic acid (ppm)	9.00	3.00	7.10
Niacin (ppm)	120.00	84.00	120.00
Pantothenic acid (ppm)	140.00	17.00	24.00
Pyridoxine (ppm)	26.00	9.60	6.00
Riboflavin (ppm)	27.00	8.10	4.50
Thiamin hydrochloride (ppm)	117.00	16.00	16.00
Vitamin A (IU/gm)	30.00	15.00	15.00
Vitamin B12 (mcg/kg)	150.00	51.00	50.00
Vitamin D3 (IU/gm)	2.00	2.30	4.50
Vitamin E (IU/kg)	135.00	99.00	42.00
Vitamin K (as menadione) (ppm)	100.00	3.30	1.30

### Two-bottle choice 24-h intermittent access (IA) alcohol consumption procedure

Mice were subjected to the IA alcohol consumption procedure as previously described (Hwa et al., 2011). Animals were given the choice of selecting either of the two bottles to drink: one containing water and the other containing 15% ethanol (V/V) (Pharmco-AAPER, Brookfield, CT) solution for 24 h every other day. On the off-days, mice had access to two bottles of water. Alcohol and water bottles were weighed prior to being placed in the cage and were weighed again 24 h later. Mice received two bottles of water over the weekend. The spillage was controlled by measuring the volume lost from alcohol and water bottles placed in an empty cage. The position of the water and alcohol bottles was switched for every drinking session. Control water-drinking mice were provided with two bottles of water. Mice were weighed once per week, and their body weights were recorded. Alcohol consumption was calculated as grams of alcohol consumed per kilogram of body weight. Alcohol preference was calculated as the ratio of the amount of alcohol consumed to the total fluid consumed per IA session.

### Blood ethanol concentration (BEC) measurement

We measured BECs using an Analox AM1 analyzer (Analox Instruments, Lunenburg, MA) as described (Avegno and Gilpin, 2019). Briefly, blood was collected from tail veins in heparinized capillaries 3 h after alcohol bottles were introduced. Blood was spun down at 9,000×*g* for 12 min, and plasma was collected. In total, 5 μL of plasma was used for BEC measurement. Single-point calibration was carried out for each set of samples with reagents provided by Analox Instruments.

#### Continuous access to sucrose, saccharin, and quinine consumption

Mice were given access to sucrose, saccharin, and quinine in a two-bottle continuous access procedure as described (Maiya et al., 2021). Mice were first given access to sucrose (4%), followed by saccharin (0.03 and 0.06%) and then quinine (100 μM, 175 μM, and 250 μM). Mice had access to each concentration of sucrose/saccharin/quinine for a total of 48 h. The positions of the tastant and water bottles were switched every 24 h. There was a 1-week interval between the different tastants when mice received two bottles of water. Bottles were weighed prior to placing them in the cage and again after 24 h to determine the amount of fluid consumed.

### Quinine adulteration of alcohol

Alcohol was adulterated with increasing concentrations of quinine ranging from 100 μM to 500 μM in the IA procedure. Mice were exposed to each concentration of quinine-adulterated alcohol for 48 h.

### Sample collection

Stool pellets were collected at two-time points: (1) immediately before the last IA session before the diet switch when mice were on the TL2019S diet and (2) immediately before the fourth session post-diet switch when mice were on the LD5001 or LD5053 diets. See Supplementary Figure S1 for a schematic of sample collection time points relative to alcohol sessions. During sample collection, mice were temporarily removed from their home cages and placed in sterile beakers. Stool pellets were collected using sterile forceps in autoclaved microtubes with attached caps and were frozen. Stool samples were sent to the Louisiana State University School of Medicine Microbial Genomics Resource Group for bacterial quantification and analysis.

### DNA extraction, PCR amplification, and sequencing

DNA extraction and sequencing were performed by the Louisiana State University School of Medicine Microbial Genomics Resource Group.^1^ The genomic DNA was extracted using the QIAamp DNA Stool Mini Kit (Qiagen, Valencia, CA), modified to include bead-beating and RNase A treatment. A negative control was set for checking any potential bacterial DNA that exists in chemicals used during the DNA extraction process.

Two steps of amplification were performed to prepare the sequencing library using the AccuPrime Taq high-fidelity DNA polymerase system (Invitrogen, Carlsbad, CA). A negative control with the control from DNA extraction and a positive control of ZymoBIOMICS™ Microbial Community Standard (ZYMO Research, Irvine, CA) were set during amplicon library preparation. The 16S ribosomal DNA hypervariable region V4 was amplified using 20 ng of genomic DNA and the gene-specific primers with Illumina adaptors: forward 5′-TCGTCGGCAGCGTCAGATGTGTATAAGAGACAGGTGCCAGCMGCCGCGGTAA-3′; reverse 5′-GTCTCGTGGGCTCGGAGATGTGTATAAGAGACAG GGACTACHVGGGTWTCTAAT-3′. The PCR conditions were as follows: 95°C for 3 min, 25 cycles of 95°C for 30 s, 55°C for 30 s, 72°C for 30 s, 72°C for 5 min, and hold at 4°C. PCR products were purified using AMPure XP beads, to which the beads were added as 0.85× PCR volume. In total, 4 μL of purified amplicon DNA from the last step was amplified eight cycles with the same PCR condition using primers with different molecular barcodes: forward 5′-AATGATACGGCGACCACCGAGATCTACAC [i5] TCGTCGGCAGCGTC-3′; reverse 5′-CAAGCAGAAGACGGCATACGAGAT [i7] GTCTCGTGGGCTCGG-3′. The indexed amplicon libraries purified using AMPure XP beads and quantified using Quant-iT PicoGreen (Invitrogen) were normalized and pooled. The pooled library was quantified using the KAPA Library Quantification Kit (Kapa Biosystems, Cape Town, South Africa) diluted and denatured as the guideline for Illumina’s sequencing library preparation. A total of 10% PhiX was added to the sequencing library as an internal control and to increase the diversity of the 16S RNA amplicon library. Paired-end sequencing was performed on an Illumina MiSeq (Illumina, San Diego, CA) using the 2 × 250 bp V2 sequencing kit. The sequencing reads were transferred to Illumina’s BaseSpace for quality analysis, and the generated raw FASTQ files were used for further bioinformatics analysis.

### Sequence analysis

Sequenced reads underwent analysis utilizing R (v4.3.1) and DADA2 (v1.26.0) (Callahan et al., 2016; Wen et al., 2023). Initial preprocessing involved the removal of 20 base pairs from both the beginning and the end of each read to eliminate low-quality regions flanking the reads. The DADA2 algorithm was then employed to identify sequence variants, with further trimming of the 5′ ends based on these variants. To enhance the detection of rare sequence variants, reads from all samples were aggregated. Taxonomic classification of sequence variants was accomplished using the Silva database (v138.1). Decontamination procedures were carried out using the decontam package (v1.20) with the prevalence method. Additionally, an abundance filter was applied, excluding sequence variants with a relative abundance below 0.01%.

Bacterial richness was estimated using three indexes: (Koob and Volkow, 2016) Observed Features represent the number of the different operational taxonomic units (OTUs) found in the sample (richness); (Glantz et al., 2020) The Shannon index considers the diversity of subspecies (richness) and the relative abundance (evenness) of each subspecies within a specific zone; and (Dubinkina et al., 2017) Simpson considers both the evenness and the percentage representation of each subspecies within a biodiversity sample in a specific zone. This index operates under the assumption that the relative proportion of individuals in an area reflects their significance to overall diversity (Nagendra, 2002).

While alpha-diversity measures the microbiome composition, beta-diversity measures the distances between the bacterial communities and the dissimilarities between them. Beta-diversity was estimated using Aitchison distance, and the centered log ratio (CLR) transformed abundances were used to perform principal component (PC) analysis. The analysis of dissimilarity (ADONIS) was performed using the permutational multivariate analysis of variance (PERMANOVA) technique, and pairwise ADONIS was applied as a *post-hoc* test (R vegan package) (By IMPACTT Investigators, 2022).

### Statistical analysis

All behavioral data are presented as the mean ± SEM. Statistical significance was assessed using one- or two-way regular or repeated measures (RM) ANOVA. *Post-hoc* Dunnett’s test was performed when a significant main effect was detected by one-way RM ANOVA. *Post-hoc* Sidak’s tests were performed when a significant interaction was detected by two-way RM ANOVA. Gut microbiome data were analyzed using analysis packages on R as described above.

## Results

### Effects of diets on alcohol consumption and preference

**Figure 1:**
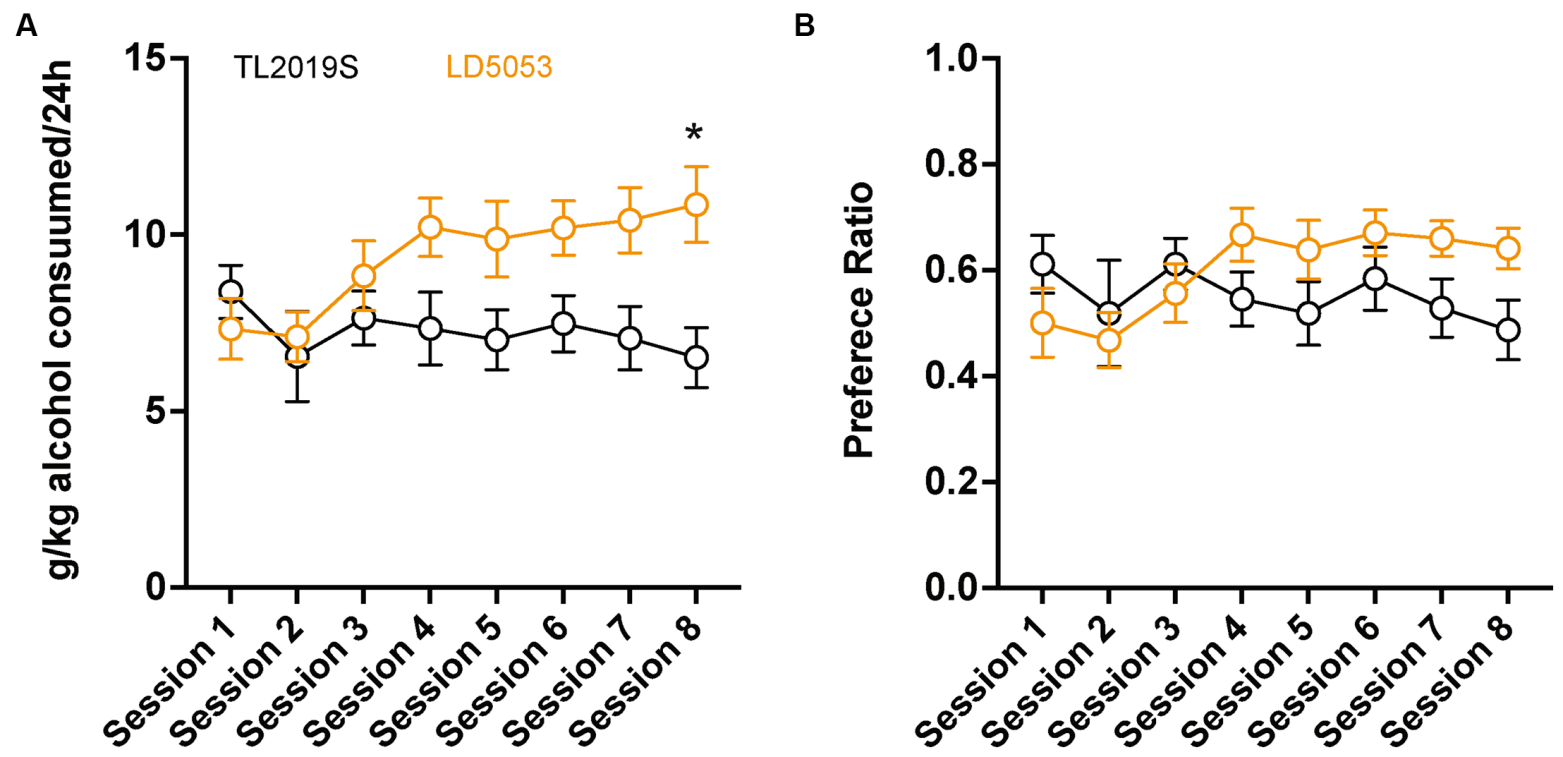
Male mice fed LD5053 consumed significantly more alcohol **(A)** and showed increased preference **(B)** for alcohol in the IA two-bottle choice procedure. *p < 0.05, Sidak’s post-test, *N* = 7/group.

We first compared alcohol consumption and preference in male C57BL/6 J mice that were maintained on LD5053 or TL2019S diets. Mice maintained on the LD5053 diet consumed significantly more alcohol than their TL2019S-fed counterparts (Figure 1A). Two-way repeated measures (RM) ANOVA of alcohol consumption revealed a significant diet × session interaction [(*F*_diet × session_ (7, 133) = 3.722, *p* = 0.0010)]. Sidak’s post-test revealed that mice on LD5053 consumed significantly higher amounts of alcohol during the last drinking session (Figure 1A). Two-way RM ANOVA of alcohol preference indicated a significant diet × session interaction (Figure 1B) [(*F*_diet × session_ (7, 133) = 6.120, *p* = 0.0061)], suggesting that alcohol preference was higher in LD5053-fed mice compared to TL2019S-fed mice during some drinking sessions. However, Sidak’s post-test did not reveal any significant differences between the two groups across any individual drinking session.

**Figure 2:**
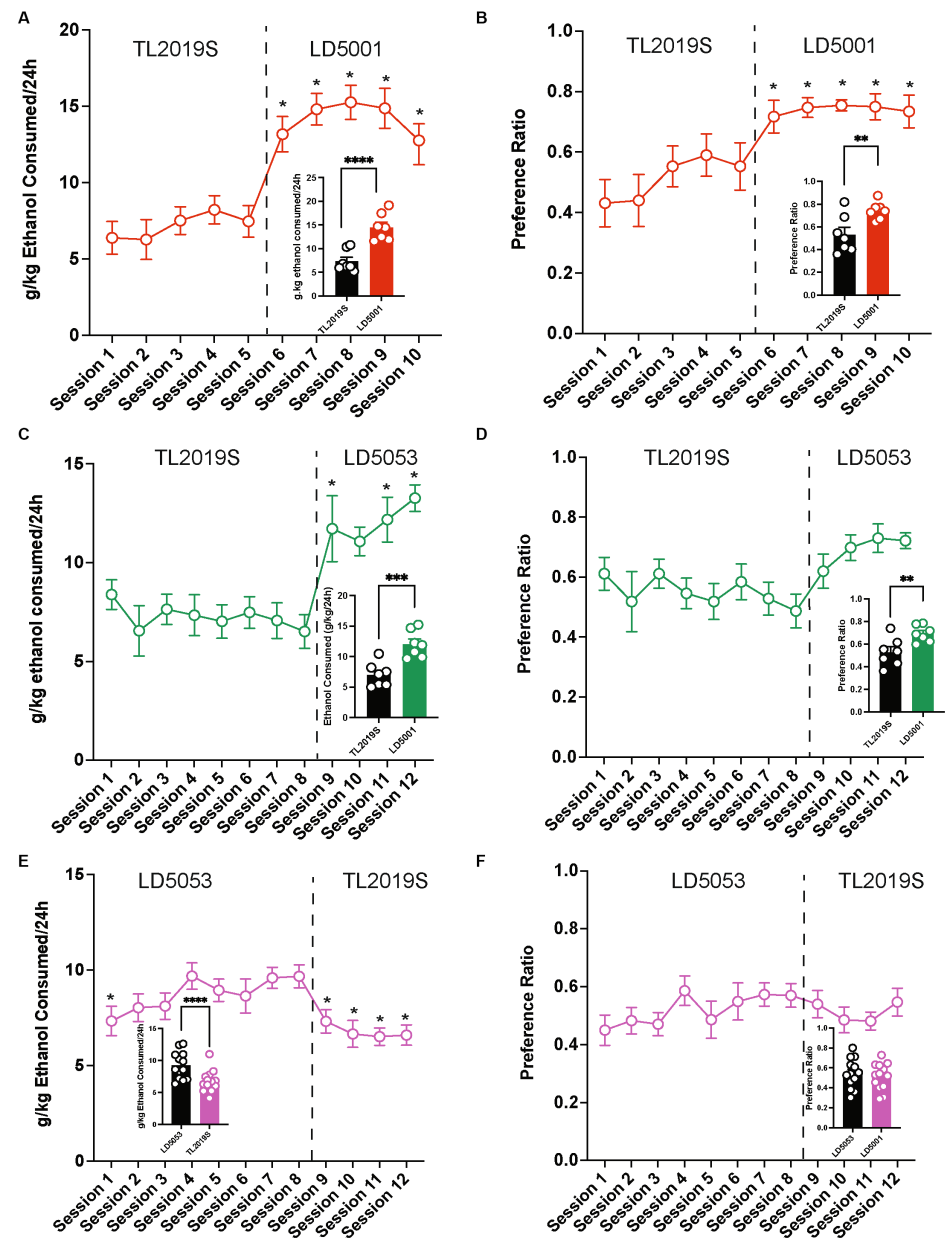
The effects of switching diets on alcohol consumption in males. **(A)** Mice consumed significantly more alcohol and showed an enhanced preference for alcohol **(B)** when switched from TL2019S to LD5001. *p < 0.05, Dunnett’s post-test compared to Session 1. LD5053 consumed more alcohol [**(A)**, inset] and showed increased alcohol preference [**(B)**, inset] compared to mice on TL2019S during the last four sessions of each diet. Student’s *t*-test, ****p < 0.0001, **p < 0.01, *N* = 7/group. Alcohol consumption **(C)** and preference **(D)** were also significantly increased when mice were switched from TL2019S to LD5053. *p < 0.05, Dunnett’s post-test compared to Session 1. The analysis of the last 4 days of each diet revealed a significant increase in alcohol consumption [**(C)**, inset] and preference [**(D)**, inset] when mice were fed LD5053. Student’s *t*-test, ***p < 0.001, **p < 0.01, *N* = 7 mice/group. Alcohol consumption and preference were significantly decreased **(E,F)** when the diet was switched from LD5053 to TJ2019S. *p < 0.05, Dunnett’s post-test compared to Session 4. A comparison of alcohol consumption and preference during the last four drinking sessions for each diet revealed a significant decrease in alcohol consumption [**(E)**, inset] but no change in preference [**(F)**, inset]. Student’s *t*-test, *p < 0.05, *N* = 7/group.

We next determined the consequence of switching the diet from TL2019S to LD5053 or LD5001, another commonly used diet formulation from Research Diets, on alcohol consumption and preference. Diets were switched 48 h prior to the alcohol-drinking session. We first measured the consequences of switching from the TL2019S to LD5001 diets. We found a significant increase in alcohol consumption (Figure 2A) and preference (Figure 2B) when mice were switched from TL2019S to LD5001. One-way RM ANOVA of alcohol consumption across sessions indicated a significant main effect of the session [*F*_session_ (9, 54) = 34.49, *p* < 0.0001]. *Post-hoc* Dunnett’s test revealed the amount of alcohol consumed during sessions 6–10 when the mice were on LD5001 was significantly higher in comparison to session 1 when the mice were fed TL20I9S. One-way RM ANOVA of alcohol preference also revealed a significant main effect of the session [*F*_session_ (9, 54) = 7.902, *p* < 0.0001]. *Post-hoc* Dunnett’s test showed that alcohol preference was significantly higher during sessions 6–10 than during session 1. We also examined average alcohol consumption (Figure 2A, inset) and alcohol preference (Figure 2B, inset) per session during the last four sessions for each diet, paired Student’s *t*-test revealed that alcohol consumption and preference were significantly enhanced when the mice were on the LD5001 diet compared to the TL2019S diet. We also examined water consumption across sessions and found no significant differences in water consumption as mice were switched from TL2019S to LD5001 diets (Supplementary Figure S2A).

Similarly, we found that switching diets from TL2019S to LD5053 also significantly increased alcohol consumption (Figure 2C) and preference (Figure 2D). One-way RM ANOVA revealed a significant main effect of the session [*F*_session_ (11, 66) = 8.834, *p* < 0.0001]. *Post-hoc* Dunnett’s test indicated that mice consumed significantly more alcohol during sessions 9, 11, and 12 when they were on LD5053 compared to session 1 when they were on TL2019S. Similarly, one-way RM ANOVA analysis of alcohol preference revealed a significant main effect of the session [*F*_session_ (11, 66) = 2.860, *p* = 0.004]. However, *post-hoc* Dunnett’s test failed to reveal any significant differences in alcohol preference in session 1 compared to other sessions. We also examined alcohol consumption and preference during the last four sessions for each diet. Paired Student’s *t*-test revealed increased alcohol consumption (Figure 2C, inset) and preference (Figure 2D, inset) when mice were fed LD5053 compared to when they were fed TL2019S. Water consumption was not significantly affected when the diet was switched from TL2019S to LD5053 (Supplementary Figure S2B).

We next examined whether mice that were drinking high amounts of alcohol on LD5053 would continue to maintain high levels of drinking when their diets were switched to TL2019S. We found that switching diets from LD5053 to TL2019S decreased alcohol consumption (Figure 2E) and preference (Figure 2F). One-way RM ANOVA analysis revealed a main effect of the alcohol-drinking session [*F*_session_ (11, 132) = 5.601, *p* < 0.0001]. *Post-hoc* Dunnett’s test revealed that mice consumed significantly lower amounts of alcohol in sessions 1, 9, 10, 11, and 12 compared to session 7 when drinking levels had stabilized. Alcohol preference was not significantly different after the diet switch compared to session 7. We also examined average alcohol consumption and preference per session during the last four sessions for each diet. We found a significant decrease in alcohol consumption when mice were switched from LD5053 to TL2019S (Figure 2E, inset). However, we did not see a significant reduction in alcohol preference during the last four sessions (Figure 2F, inset).

**Figure 3:**
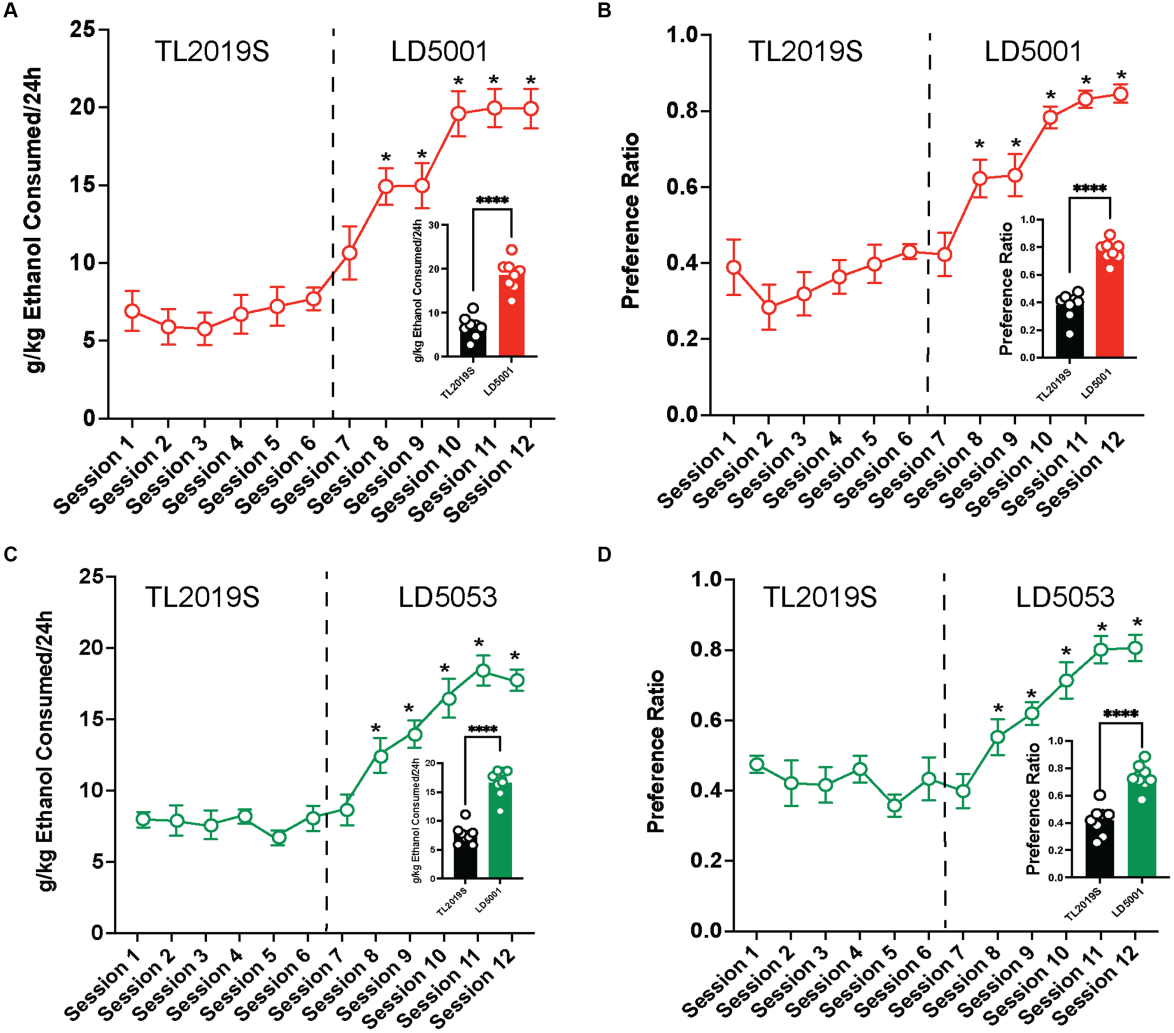
The effects of switching diets on alcohol consumption in females. Female C57BL/6 J mice consumed significantly more alcohol **(A)** and showed increased preference **(B)** for alcohol when the diet was switched from TL2019S to LD5001. The analysis of alcohol consumption and preference during the last 4 days on each diet also revealed that female mice consumed more alcohol [**(A)**, inset] and showed increased preference [**(B)**, inset] for alcohol when fed LD5001. Student’s *t*-test, ****p < 0.0001, *N* = 8/group. Female mice also consumed significantly **(C)** more and showed enhanced preference **(D)** for alcohol when fed LD5053 than when fed TL2019S. The analysis of alcohol consumption during the last four sessions for each diet revealed that mice consumed significantly more alcohol [**(C)**, inset] and showed more preference for alcohol [**(D)**, inset] when fed LD5053 compared to mice fed TL2019S. Student’s *t*-test, ****p < 0.0001, *N* = 8/group.

We next examined whether these diet influences on alcohol intake could be extended to females. We found that switching diets from TL2019S to LD5001 significantly increased alcohol consumption (Figure 3A) and preference (Figure 3B) in female mice. One-way RM ANOVA indicated a significant main effect of drinking sessions [*F*_session_ (11, 77) = 32.2, *p* = 0.0001]. *Post-hoc* Dunnett’s test revealed that mice consumed significantly more alcohol in sessions 8–12 when they were on LD5001 compared to session 1 when they were on the TL2019S diet. One-way RM ANOVA of alcohol preference also revealed a significant main effect of drinking session [*F*_session_ (11, 77) = 20.37, *p* = 0.0001]. *Post-hoc* Dunnett’s tests indicated that female mice displayed an enhanced preference for alcohol in sessions 8–12 compared to session 1. We also compared average alcohol consumption and alcohol preference during the last four sessions for each diet. We found that mice consumed significantly more alcohol (Figure 3A, inset) and showed significantly increased alcohol preference (Figure 3B, inset) when they were on LD5001. We also measured water consumption across sessions and found that, in contrast to male mice, female mice consumed significantly less water when switched from TL2019S to LD5001 (Supplementary Figure S2C). One-way RM ANOVA revealed a main effect of the session [*F*_session_ (11, 77) = 11.20, *p* < 0.0001]. *Post-hoc* Dunnett’s test revealed that water consumption was significantly lower for sessions 11 and 12 compared to session 1.

Similarly female mice consumed significantly more (Figure 3C) and showed enhanced preference (Figure 3D) for alcohol when switched from TL2019S to LD 5053. One-way RM ANOVA revealed a significant main effect of drinking session [*F*_session_ (11, 77) = 32.56, *p* = 0.0001]. *Post-hoc* Dunnett’s test revealed that females consumed significantly more alcohol during sessions 8–12 when they were on LD5053 compared to session 1 when they were on the TL2019S diet. One-way RM ANOVA of alcohol preference also indicated a significant main effect of the drinking session [*F*_session_ (11, 77) = 20.09, *p* = 0.0001]. *Post-hoc* Dunnett’s test revealed that mice drank significantly more alcohol during sessions 8–12 when compared to session 1. Examining the average alcohol consumption during the last four sessions for each diet revealed that female mice on LD5053 consumed significantly more alcohol (Figure 3C, inset) and showed significantly enhanced preference for alcohol (Figure 3D, inset) during the last four sessions. Water consumption was also significantly reduced when the diet was switched from TL2019S to LD5053 (Supplementary Figure S2D). One-way RM ANOVA revealed a main effect of the session [*F*_session_ (11, 77) = 9.776, *p* < 0.0001]. *Post-hoc* Dunnett’s test revealed that water consumption was significantly higher for session 7 and significantly lower for sessions 11 and 12 compared to session 1.

We also examined body weight when the mice were on different diets. Average body weights did not differ significantly between males maintained on TL2019S or LD5001 (Supplementary Figure S3A). There was a small but statistically significant increase in body weight in mice maintained on LD5053 compared to those maintained on TL2019S (Supplementary Figure S3B, Student’s *t*-test, *p* < 0.001). There were no significant differences in body weights between females maintained on TL2019S and LD5001 (Supplementary Figure S3C) or LD5053 (Supplementary Figure S3D).

**Figure 4:**
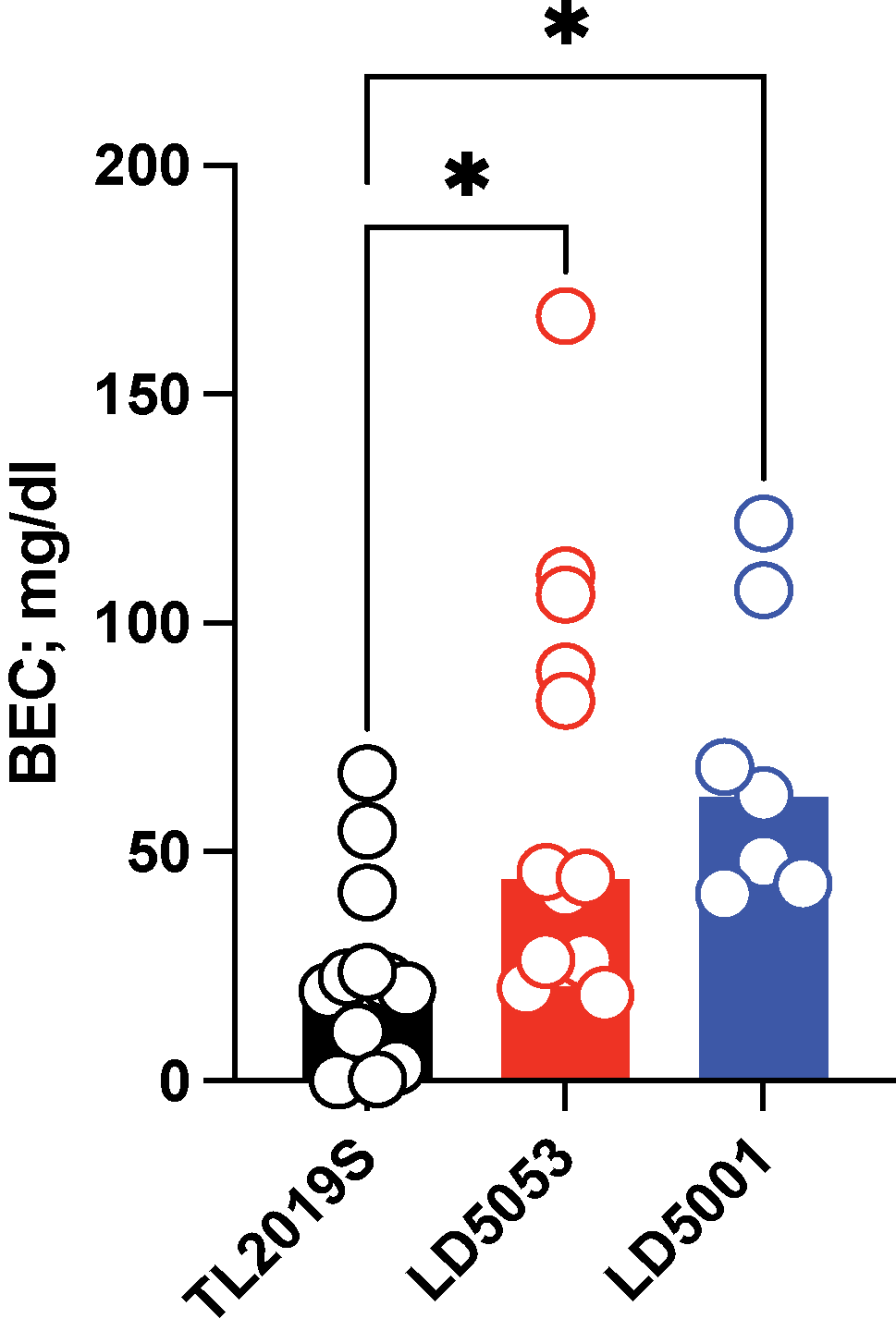
Significantly higher BECs were detected in male mice fed LD5001 and LD5053-fed mice compared to TL2019S. *p < 0.05, Dunnett’s test, *N* = 7–13/group.

We next examined blood ethanol concentrations from male mice that were maintained on the three different diets. Tail vein blood was collected 3 h after alcohol bottles were introduced. One-way ANOVA revealed a significant main effect of diet on alcohol consumption (Figure 4) [*F*_diet_ (2, 30) = 5.723, *p* = 0.0078]. Dunnett’s *post-hoc* test indicated that mice on LD5053 and LD5001 showed significantly higher BECs than mice on TL2019S, which is consistent with these mice consuming higher amounts of alcohol.

### Effects of diets on sweet and bitter taste perception and quinine-resistant drinking

**Figure 5:**
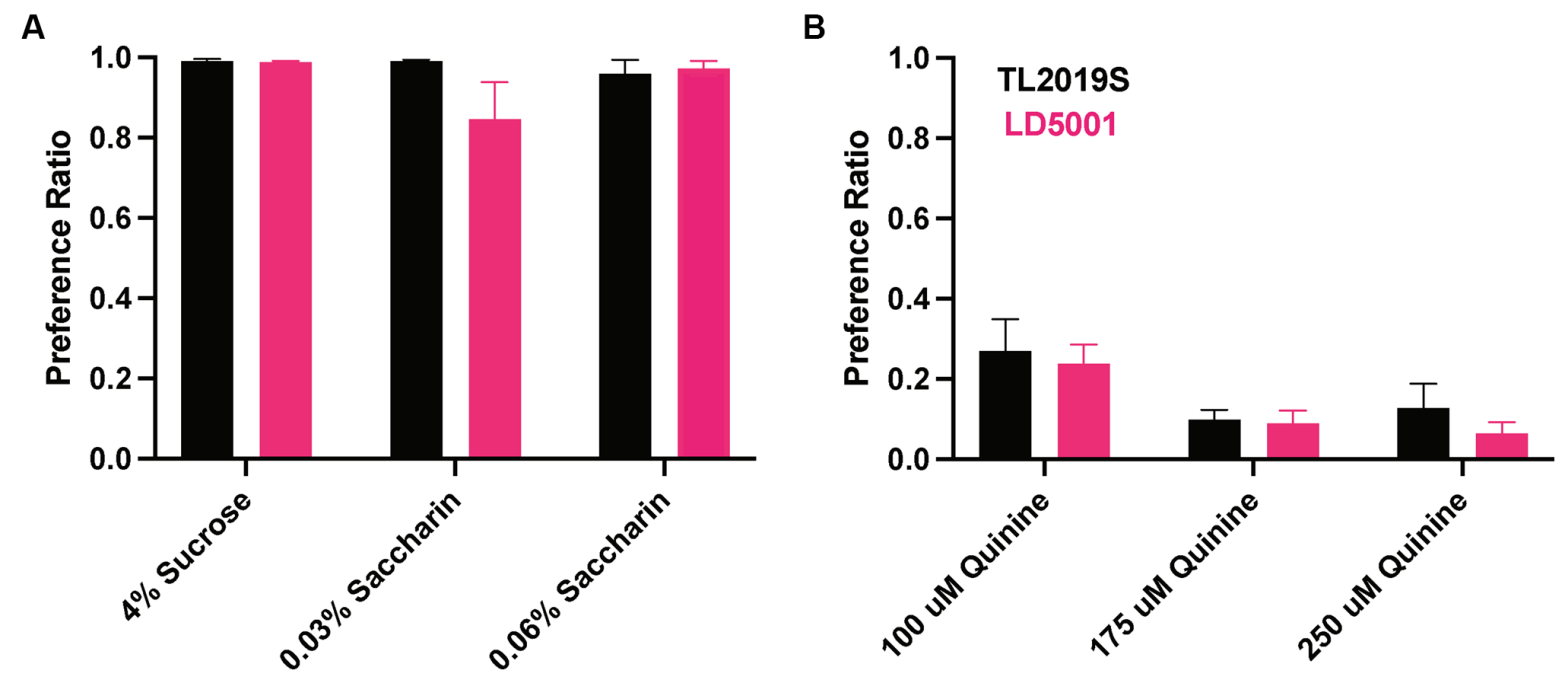
Taste perception was unaltered in male mice consuming LD5001. Sucrose, saccharin preference **(A)**, and quinine preference **(B)** were unaltered in mice fed with LD5001 compared to TL2019S. *N* = 9–11/group.

We focused on LD5001 diet for future experiments, as mice that were fed this diet consumed higher relative amounts of alcohol and had higher BECs. We next determined whether the increased alcohol consumption observed with LD5001 was due to altered sweet and bitter taste perception. Therefore, we examined sucrose (4%), saccharin (0.04 and 0.06%), and quinine consumption (100, 175, and 200 μM) in male mice maintained on the LD5001 or TL2019S diets. We first examined sucrose consumption, followed by consumption of increasing concentrations of saccharin and quinine. We found no significant differences in sucrose, quinine, or saccharin consumption between mice that were on TL2019S compared to those that were on LD5001 (Figure 5), suggesting that the increase in alcohol consumption observed in mice that were fed LD5001 is unlikely due to altered sweet and bitter taste perception.

**Figure 6:**
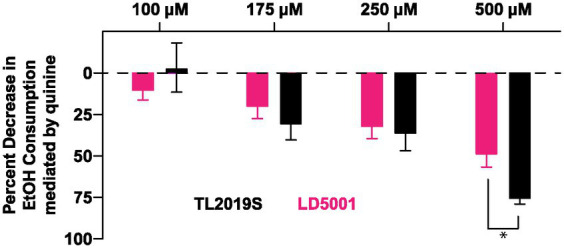
Diet-induced increase in alcohol consumption is resistant to quinine adulteration. Quinine adulteration reduced alcohol consumption in both LD5001- and TL2019S-fed male mice. However, at the highest and most aversive dose of quinine (500 μM), LD5001-fed mice displayed more resistance to quinine adulteration and maintained high levels of intake. *p < 0.05, Sidak’s post-test. *N* = 7/group.

We next determined whether alcohol consumption in LD5001-fed mice was sensitive to disruption by quinine adulteration. We added increasing concentrations of quinine to the alcohol. Quinine adulteration dose-dependently decreased alcohol consumption in both TL2019S and LD5001-fed mice (Figure 6). However, at the highest and most aversive concentration of quinine tested, the magnitude of decrease in drinking was greater in TL2019S-fed mice than in mice fed LD5001, indicating that LD5001 led to a higher degree of quinine resistance in these mice (Figure 6). Two-way RM ANOVA revealed a significant main effect of quinine concentration [*F*_quinine_ (3, 36) = 24.02, *p* < 0.0001] and a quinine × diet interaction [*F*_quinine × diet_ (3, 36) = 2.902, *p* = 0.0481]. *Post-hoc* Sidak’s test indicates that mice that were fed the TL2019S diet consumed significantly less alcohol when it was adulterated with 500 μM of quinine than mice on the LD5001 diet.

### Effects of standard rodent diets on gut microbiome diversity

**Figure 7:**
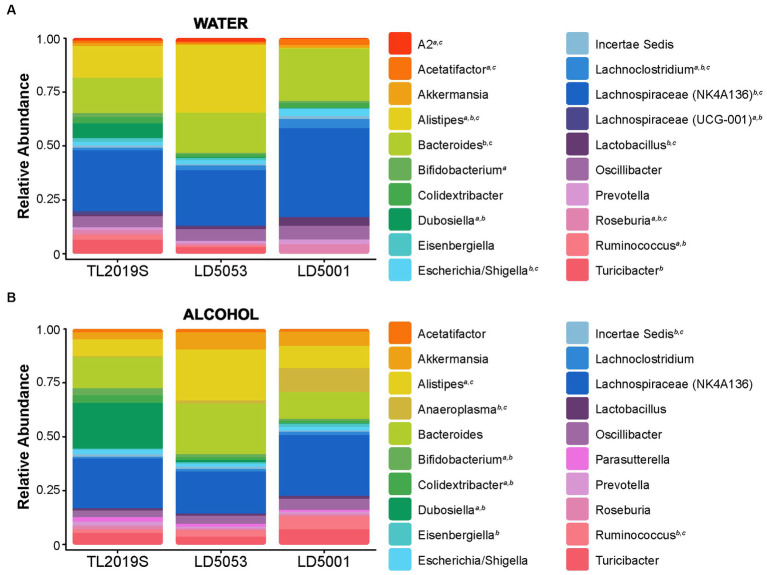
Rodent diets shape gut bacterial abundance. The relative abundances were estimated for the three diets during the water-drinking period **(A)** and after the alcohol sessions **(B)**. *N* = 7/group. Stool samples were collected at two-time points: (1) immediately before the last session of alcohol-drinking on the TL2019S diet and (2) immediately before the fourth alcohol-drinking session after the diet switch when mice are on the LD5001 or LD5053 diet. Samples were also collected from a separate cohort of water-drinking mice that were exposed to the diets for similar time points.

We next measured the effects of diet on gut microbiome alpha-diversity in male mice. Alpha-diversity measures the ecological diversity within the sample, and it can be subdivided into evenness and richness. Evenness, or relative abundance, is a measure of the sequence variant uniformity, while sample richness represents the number of each operational taxonomic unit (OTU) present in the sample. We assessed alpha-diversity of the stool samples from mice fed TL2019S, LD5053, and LD5001 diets. Stool samples were collected from alcohol-drinking mice that were part of the diet-switching experiment as shown in Figure 2. Mice were switched from TL2019S to LD5053 or LD5001. Samples were collected at two-time points: (1) immediately before the last session of alcohol-drinking on the TL2019S diet and (2) immediately before the fourth alcohol-drinking session after the diet switch when mice are on the LD5001 or LD5053 diets. Samples were also collected from a separate cohort of water-drinking mice that were exposed to TL2019S (10 days) and LD5001 and LD5053 (9 days) for similar amounts of time. Figure 7A shows the effect of diet on relative abundance at the genus level in the water-drinking group. Two-way ANOVA of relative abundance in water-drinking animals revealed a significant diet × genera interaction [F_diet × genera_ (38, 360) = 18.67, *p* = 7.84E-64]. *Post-hoc* Tukey test showed that mice on the TL2019S diet had a significantly higher abundance of *Dubosiella* (6.9%) compared to those on LD5053 (0.1%) and LD5001 (0%) (*p* = 0.0041). Animals on LD5053 showed an increased relative abundance of the genus *Alistipes* (31.6%) compared to TL2019S (14.7%) and LD5001 (0.2%) (*p* < 0.0001, Tukey test). In contrast, animals on LD5001 had a higher relative abundance of the genus *Lachnospiraceae* NK4A136 group (41.4%) than TL2019S (28.4%) and LD5053 (25.8%) (*p* = 0.0047, Tukey test) and higher relative abundance of *Bacteroides* (24.5%) compared to TL2019S (16.6%) and LD5053 (18.5%) (*p* = 0.0349, Tukey test). The overall relative abundance comparisons for the water-drinking group are compiled in Supplementary Table S2A.

We next assessed bacterial relative abundance in samples from animals after IA alcohol consumption (Figure 7B). Two-way ANOVA analysis of relative abundance in alcohol-drinking animals revealed a significant diet × genera interaction [*F*_diet × genera_ (38, 360) = 8.91, *p* = 5.62E-33]. Alcohol-drinking mice on TL2019S diet showed a higher relative abundance of *Dubosiella* (21.4%) compared to LD5053 (1%) and LD5001 (0%) (*p* < 0.0001, Tukey test). Animals on LD5053 diet had an increased relative abundance of *Alistipes* (23.8%) in comparison to TL2019S (8.1%) and LD5001 (10.3%) (*p* = 0.0008, Tukey test). LD5001 has a higher relative abundance of the genus *Anaeroplasma* (11%) compared to TL2019S (0.02%) and LD5053 (1.1%) (*p* = 0.0008, Tukey test). The overall relative abundance comparisons for the alcohol-drinking group are compiled in Supplementary Table S2B.

**Figure 8:**
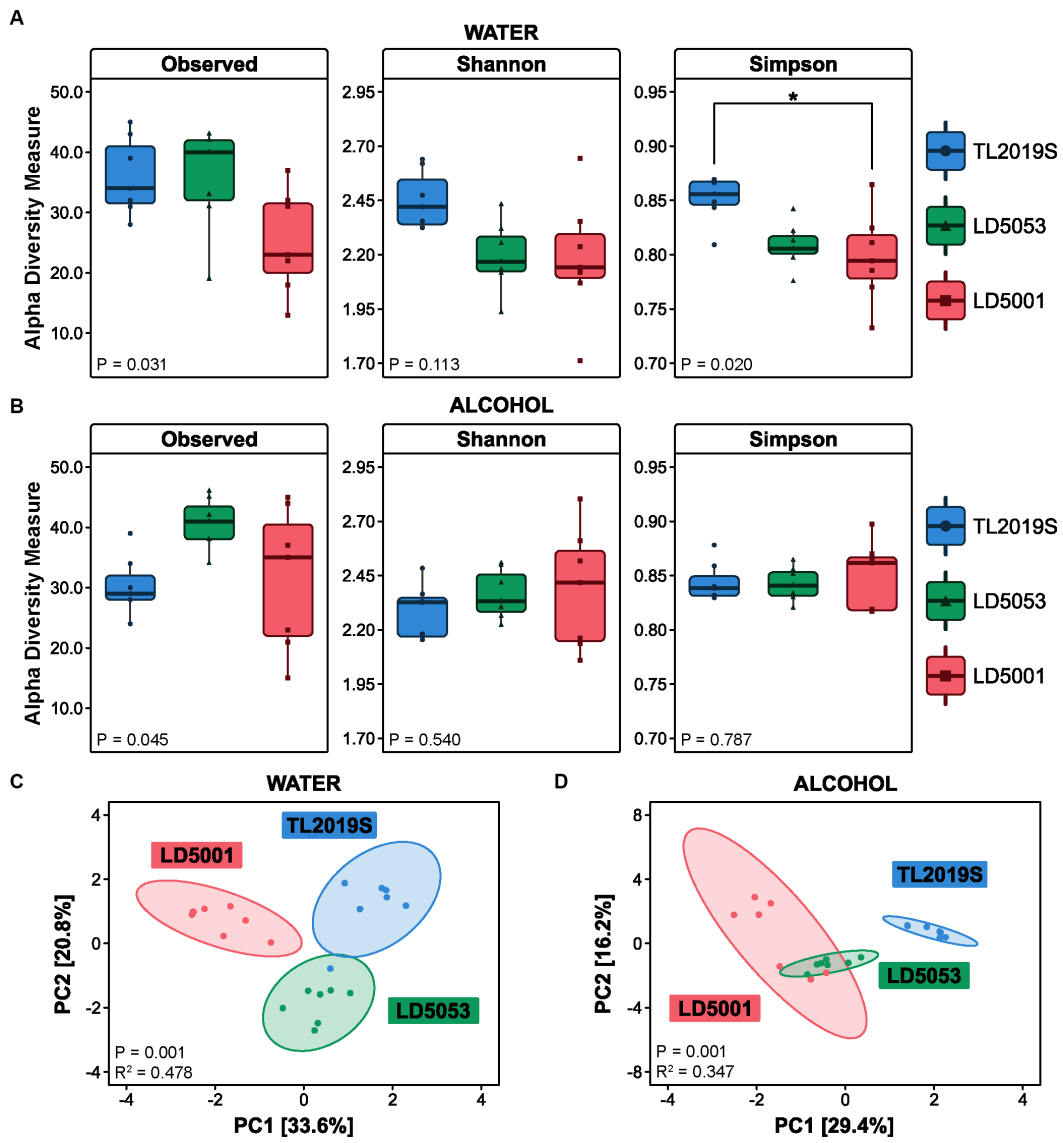
Gut microbial structure is influenced by different rodent diets. Bacterial counts were transformed into a centered log ratio (CLR) and used to estimate alpha- and beta-diversities (*N* = 7/group). Alpha-diversity was assessed using Observed Features, Shannon, and Simpson indices. **(A)** During the water-drinking sessions, the ANOVA test revealed significant differences in Observed Features (*p* = 0.031) and the Simpson index (*p* = 0.020); the *post-hoc* Tukey test only identified significance in the Simpson index between Teklad and LD5001 diets (*p* = 0.026). **(B)** After the intermittent access alcohol (IA) protocol, we did not find significant differences in alpha-diversity. Beta-diversity was calculated in terms of Euclidean distance using the centered log ratio (CLR) values. PERMANOVA (ADONIS) results were depicted here as a principal component (PC) graph. **(C)** Beta-diversity was significantly changed in the water-drinking group (PERMANOVA [*p* = 0.001, *R*^2^ = 0.478]); *post-hoc* pairwise ADONIS tests revealed significant differences between TL2019s vs. LD5053 (*p* = 0.009), TL2019S vs. LD5001 (*p* = 0.006), and LD5053 vs. LD5001 (*p* = 0.006). **(D)** Animals in the IA group also presented significant changes in beta-diversity PERMANOVA (*p* = 0.001, *R*^2^ = 0.347); *post-hoc* pairwise ADONIS tests revealed significant differences between TL2019S vs. LD5053 (*p* = 0.006), TL2019S vs. LD5001 (*p* = 0.003), and LD5053 vs. LD5001 (*p* = 0.006).

Sample richness was significantly different between the three different diets in the water-drinking group (Figure 8A). One-way ANOVA revealed significant differences in Observed Features [*F*_diet_ (2, 18) = 4.22, *p* = 0.0313]. However, Tukey’s post-test did not show statistical differences between the groups: TL2019S vs. LD5053 (*p* = 0.9975), TL2019S vs. LD5001 (*p* = 0.0502), and LD5053 vs. LD5001 (*p* = 0.0573). No differences were found on the Shannon index [*F*_diet_ (2, 18) = 2.463, *p* = 0.1134]. The Simpson index was significantly different between the groups [*F*_diet_ (2, 18) = 4.877, *p* = 0.0203]. Tukey’s multiple comparison showed significant differences among the groups TL2019S vs. LD5001 (mean difference *p* = 0.0261; 95% CI = [0.005722–0.09656]). No significant differences were found between TL2019S and LD5053 (*p* = 0.0558) or between LD5053 and LD5001 (*p* = 0.9249). Bacterial richness was evaluated in samples from the three diet groups in alcohol-drinking animals (Figure 8B). One-way ANOVA shows that the three diet groups have distinct Observed Features [*F*_diet_ (2, 18) = 3.691, *p* = 0.0454]. No statistical differences were found on the multiple comparison tests: TL2019S vs. LD5053 (*p* = 0.0977), TL2019S vs. LD5001 (*p* = 0.9591), and LD5053 vs. LD5001 (*p* = 0.0578). Both Shannon and Simpson indices did not show significant statistical differences between the three different diets after alcohol intake.

We sought to investigate whether the three different diets would result in greater dissimilarities between the bacterial communities using beta-diversity metrics. Beta-diversity was calculated using the Aitchison distance, which is the Euclidean distance between data transformed by the centered log ratio (CLR) method. Water-drinking mice presented diet-dependent differences in the variation of the communities’ composition. The variance explained by component 1 was 33.6% and by component 2 was 20.8% (Figure 8C). ADONIS test revealed a statistically significant difference among the three diets [*F*_diet_ (2, 18) = 8.24, *p* = 0.001]. *Post-hoc* pairwise ADONIS tests revealed significant differences between TL2019s vs. LD5053 (*p* = 0.009), TL2019S vs. LD5001 (*p* = 0.006), and LD5053 vs. LD5001 (*p* = 0.006). The beta-diversity values were also significantly different between the alcohol-drinking groups [*F*_diet_ (2, 18) = 4.77, *p* = 0.001] (Figure 8D). *Post-hoc* pairwise ADONIS tests revealed significant differences between TL2019S vs. LD5053 (*p* = 0.006), TL2019S vs. LD5001 (*p* = 0.003), and LD5053 vs. LD5001 (*p* = 0.006).

## Discussion

Our results indicate that mice fed LD5001 and LD5053 consume more, show an enhanced preference for alcohol, and attain higher BECs compared to mice fed TL2019S. Sucrose, saccharin, or quinine consumption was not affected by different brands of chow, suggesting that the increase in alcohol consumption observed with LD5001 and LD5053 was not due to altered sweet and bitter taste perception. Furthermore, alcohol consumption in mice fed LD5001 was more resistant to quinine adulteration when compared with mice that were fed TL2019S. Gut microbiome analysis revealed that the gut bacteria in water- and alcohol-drinking mice fed LD5001, LD5053, and TL2019S significantly differed in terms of alpha- and beta-diversity and displayed distinct gut microbial structures.

### Standard rodent diets differentially impact alcohol consumption and preference

Previous studies have examined the impact of several commercially available rodent diet formulations on alcohol consumption. One recent study examined binge alcohol consumption in rodents fed LD5001 or TL2920S and found that alcohol consumption and BECs were markedly higher in mice maintained on LD5001 compared to those on TL2920S (Maphis et al., 2022). Mice on LD5001 also displayed increased front-loading behavior and consumed twice as much alcohol in the first 15 min than TL2920S-fed mice (Maphis et al., 2022). Another recent study compared mice maintained on four different commercially available rodent diets—LD5001, H7012, H2918, and LDV575—on IA alcohol consumption. They found that mice maintained on the LD5001 and H7012 diets consumed high amounts of alcohol compared to the other two diets (Quadir et al., 2020). A previous study examined the effects of six commercially available rodent diets on alcohol consumption in the “Drinking in the dark” model of binge alcohol consumption and continuous access to two-bottle choice drinking. The diets evaluated were RMH3000 (Purina) and Teklad diets T. 2,916, T.2918. T.2920x, T.7912, and T.8940. Mice maintained on T.7912 consumed the highest amount and showed the largest preference for alcohol (Marshall et al., 2015). However, none of these studies have examined the mechanisms by which these different diets influence alcohol intake.

Consistent with these previously published findings, we found that standard rodent diet formulations can profoundly and differentially influence alcohol consumption in mice. The three diets we tested, namely, LD5001, LD5053, and TL2019S, have numerous differences in macro- and micronutrient compositions (Table 1). The diets differed in carbohydrate, fat, and protein contents. LD5053 and LD5001 had higher protein and carbohydrate content than TL2019S. Notably, TL2019S had higher fat content than LD5001 and LD5053. While body weights did not change with the three diets, it is possible that body composition may have changed, which could in turn influence alcohol metabolism and alcohol consumption (Liangpunsakul et al., 2010). There were also significant differences in the amounts of vitamins and minerals between the diets, with higher amounts of vitamins A, E, K3, B1, and B2 in TL2019S compared to LD5001 and LD5053. Higher amounts of calcium, phosphorous, potassium, chloride, and magnesium were present in TL2019S compared to LD5001 and LD5053. There were also higher amounts of sulfur, cobalt, fluorine, and chromium in LD5001 and LD5053 compared to TL2019S. There were also textural differences between LD5053 and LD5001 compared to TL2019S, with TL2019S being grittier in texture than the LD diets. The difference in texture might result from a variety of reasons, including the method employed to sterilize these foods. TL2019S is sterilized by autoclaving, whereas LD5001 and LD5053 are gamma-irradiated. It is unclear which of these differences contribute significantly to increased alcohol consumption.

One recent meta-analysis examined the concentration of isoflavones in various commercially available rodent diet formulations and found that they may vary by as much as 20–600 mg/g of diet. There is a significant positive correlation between alcohol consumption, preference, and isoflavone concentration in male mice (Eduardo and Abrahao, 2022). We do not know the isoflavone concentrations in the rodent diets that were used in our study, and hence, it remains to be determined whether isoflavones underlie the differences observed in our study.

Although we did not directly measure the amount of food consumed in our study, we found no differences in body weight when the mice were maintained on different diets, which suggests that perhaps food intake was not significantly different.

There was a trend toward reduced water consumption in males maintained on LD5053 and LD5001 that did not reach statistical significance. Water intake was significantly reduced in females maintained on both LD5001 and LD5053 compared to mice maintained on TL2019S. One possible reason for this could be that in females, water consumption was reduced to compensate for the increased amounts of alcohol consumed.

We did not find any diet-induced changes in sucrose, saccharin, or quinine preference in our study. We chose to examine the effects of diets on only sweet and bitter taste perception because there is evidence in both humans (Lanier et al., 2005; Kurshed et al., 2022) and rodents (Blednov et al., 2008; Brasser et al., 2010; Koh et al., 2024) that these two taste modalities are strongly associated with alcohol consumption and preference. This finding contrasts with a previous study suggesting diet formulations that increase alcohol consumption can also increase sucrose/saccharin consumption (Marshall et al., 2015). One limitation of this study is that we did not measure diet influences on salt, bitter, sour/acid, and fatty acid taste perception. An interesting finding in our study is that the diet that resulted in relatively higher amounts of alcohol consumption, LD5001, also resulted in significant resistance to quinine adulteration of alcohol compared to mice on the TL2019S diet. Quinine-resistant alcohol intake is frequently used to model drinking despite aversive consequences (De Oliveira et al., 2023). It is important to highlight that both TL2019S- and LD5001-fed mice decreased their drinking when alcohol solution was adulterated with increasing concentrations of quinine. However, the magnitude of this reduction was greater in TL2019S-fed mice than in LD5001-fed mice at the highest concentration of quinine tested, suggesting increased resistance to quinine adulteration in LD5001-fed mice. These results suggest that rodent diet formulation can influence the development of compulsive alcohol-seeking in mice.

### Standard rodent diets differentially impact gut microbiome diversity

To determine the mechanism by which diet could influence alcohol intake, we examined the gut microbiota of water- and alcohol-drinking mice maintained on different diets. Diet can profoundly influence gut microbial composition (Tuck et al., 2020), which can, in turn, influence alcohol consumption and reward via multiple mechanisms, including signaling through the gut–brain axis (Leclercq et al., 2019, 2020; Salavrakos et al., 2021; Quoilin et al., 2023). In fact, a recent study showed that fecal microbiota transplants from healthy donors can reduce alcohol preference and craving in people with AUD, and this behavior is transmissible to germ-free mice (Wolstenholme et al., 2022).

Animals fed with TL2019S had lower alcohol preference and consumption and higher abundance of the genus *Dubosiella* compared to the two other diets. Interestingly, *Dubosiella* was previously associated with the production of beneficial metabolites, such as short-chain fatty acids (SCFA) (Kadyan et al., 2023). SCFAs, such as acetate, butyrate, and propionate, supplementation are effective in reducing stress-induced gut–brain axis disorders (van de Wouw et al., 2018). The higher abundance of *Dubosiella* and its metabolites might be associated with lower motivation to alcohol-drinking. The genus *Alistipes* was markedly increased in water-drinking mice that were fed LD5053, and this characteristic was maintained after alcohol exposure. Prior research demonstrated a high abundance of this genus in mice subjected to stress (Bangsgaard Bendtsen et al., 2012) and in patients diagnosed with depression (Naseribafrouei et al., 2014). *Alistipes* hydrolyze tryptophan to produce indole. Tryptophan is also a precursor for serotonin, so a higher abundance of *Alistipes* could indirectly reduce serotonin availability in the gut, impairing the gut–brain axis signaling (Vlainić et al., 2016), which could in turn influence alcohol consumption and preference.

The gut microbiome of LD5001-fed mice showed the greatest changes in relative abundance after alcohol consumption. In water-drinking mice maintained on LD5001, *Lachnospiraceae* NK4A136 and *Bacteroides* were the predominant genera. The relative abundance of these genera was significantly higher in water-drinking but not alcohol-drinking LD5001-fed mice compared to TL2019S- and LD5053-fed mice. One possibility is that the increased abundance of this genera is associated with the initiation of increased alcohol consumption behavior. It is possible that other bacterial genera/networks may be important for the maintenance of continued high alcohol-drinking behavior. Increased abundance of *Lachnospiraceae* NK4A136 is associated with elevated stress, anxiety, and depression-like behaviors (Pizarro et al., 2021). Additionally, the abundance of this bacteria predicts lower concentrations of serotonin in the prefrontal cortex (PFC) (Wong et al., 2016; McGaughey et al., 2019). It is possible that the increased alcohol consumption we observe in mice fed LD5001 could be due to increased anxiety-like behaviors, although we did not test for anxiety-like behaviors in this study. *Bacteroides* is a genus of commensal microbes, and it is usually shown to be depleted after alcohol exposure (Xiao et al., 2018; Addolorato et al., 2020); however, the association of *Bacteroides* and alcohol-seeking behavior is not known and needs to be further investigated. Future studies will use bioinformatics strategies to determine bacterial networks that are significantly correlated with diets that result in increased alcohol consumption.

One caveat with our study is that, unlike alcohol-drinking mice, water-drinking mice were maintained on TL2019S, LD5053, or LD5001 and were not subject to diet switching. Hence, it is possible that some of the changes in bacterial diversity observed in alcohol-drinking mice after the diet switch may not be observed in mice that were maintained on a single type of diet throughout the experiment. However, since we waited almost 9 days after the diet switch to collect stool samples from alcohol-drinking mice, the gut microbiome could have stabilized at this time point and (David et al., 2014) be largely representative of the diet post-switch.

Gut microbiota could modulate alcohol consumption in a variety of ways, including by altering neuroinflammation, myelin synthesis, blood–brain barrier permeability, and the production of metabolites that alter signaling along the gut–brain axis (Leclercq et al., 2014, 2019). In this study, we did not perform metabolomics studies to determine whether the different diets altered the metabolic profile of the gut microbiota. The goal of this study was limited to identifying bacterial genera that could account for diet-induced differences in alcohol consummatory behaviors. Hence, one limitation of this study is that we did not establish causal relationships between specific bacterial genera and alcohol consumption. Future studies will employ techniques, such as fetal microbial transplant in germ-free mice and metabolomics, to determine specific bacterial genera and bacterial metabolites that mediate changes in alcohol intake.

Emerging findings reveal the existence of peripheral mechanisms capable of modulating reward-seeking behaviors previously attributed solely to the central nervous system. Food intake modulation can elicit effects reminiscent of those induced by addictive substances such as ethanol and nicotine, which directly enhance VTA neuron firing (Juarez and Han, 2016). Gut stimulation with caloric nutrients prompts significant striatal dopamine (DA) release. Mice consuming high-fat diets fail to display the calorie-dependent DA efflux seen in their low-fat diet counterparts. However, this deficiency in high-fat diet-induced DA release can be corrected by the dietary satiety mediator oleoylethanolamine (Ren et al., 2010; de Araujo et al., 2012; Ferreira et al., 2012). Similarly, it is possible that therapies targeting the gut microbiome, such as probiotics and fecal microbiota transplant, could be viable approaches to treat substance use disorders (Fuenzalida et al., 2021; Pizarro et al., 2021; Wolstenholme et al., 2024).

In summary, our results provide strong evidence that standard rodent diet formulations can profoundly influence alcohol consumption, preference, and compulsive alcohol intake. Hence, it is imperative that studies examining voluntary alcohol consumption document the type of rodent diet that the mice were maintained on to increase reproducibility across labs. Importantly, our result also suggests that commercially available rodent diet formulations can profoundly and differentially impact gut microbiome diversity, which could contribute to regulating alcohol consumption behaviors.

## Data availability statement

Microbiome data generated in this study are deposited in the NCBI sequence read archive, BioProjectID: PRJNA1078830, available at: https://www.ncbi.nlm.nih.gov/bioproject/?term=PRJNA1078830.

## Ethics statement

The animal study was approved by the LSUHSC Institutional Animal Care and Use Committee. The study was conducted in accordance with the local legislation and institutional requirements.

## Author contributions

AZ: Conceptualization, Formal analysis, Investigation, Methodology, Project administration, Supervision, Writing – original draft, Writing – review & editing, Visualization. ED: Formal analysis, Methodology, Visualization, Writing – review & editing, Validation. SW: Formal analysis, Methodology, Writing – review & editing, Data curation, Investigation. FP: Data curation, Formal analysis, Investigation, Methodology, Writing – review & editing. CG: Data curation, Formal analysis, Investigation, Writing – review & editing. KC: Data curation, Formal analysis, Investigation, Writing – review & editing. SB: Formal analysis, Investigation, Writing – review & editing. EG: Investigation, Writing – review & editing, Data curation. RV: Investigation, Writing – review & editing. ML: Investigation, Writing – review & editing, Data curation. CT: Investigation, Writing – review & editing, Formal analysis, Supervision. DW: Supervision, Writing – review & editing. RM: Supervision, Writing – review & editing, Conceptualization, Data curation, Formal analysis, Funding acquisition, Investigation, Methodology, Project administration, Resources, Writing – original draft.

## Footnotes

^1^ http://metagenomics.lsuhsc.edu/
