# Supplementary material for: Standard rodent diets differentially impact alcohol consumption, preference, and gut microbiome diversity
Source: Front Neurosci. 2024 May 13;18:1383181. doi: 10.3389/fnins.2024.1383181 (PMC11129685; doi:10.3389/fnins.2024.1383181)
Supplement: Supplementary file 1 [file Table_1.DOCX]

**Figure S1:** Schematic depicting timeline of diet switch experiments and sample collection timepoints for gut microbiome analysis. Timeline of A) TL2019S – LD5001, B) TL2019S – LD5053 and LD5053 – TL2019S, and TL2019S – LD5001/LD5053 diet switch experiments.

**Figure S2:** The effects of switching diets on water consumption. There were no significant differences in water consumption when diet was switched from TL2019S to LD 5001 **(A)** or LD5053 **(B)** in males. N =7/group. In females, water consumption was significantly reduced when diet was switched from TL2019S to LD5001. *, P<0.05, Dunnett’s post-test compared to Session 1 **(C)**. Water consumption was also significantly reduced when diet was switched from TL2019S to LD5053 diet **(D)**. *, P<0.05, Dunnett’s post-test compared to session1. N =8/group.

**Figure S3**: Average bodyweights across sessions during the diet switching experiment. Bodyweights did not significantly differ in male mice when they were maintained on LD5001 when compared to when they were maintained on LD 5053 **(A)**. There was a small but significant increase in body weight in mice maintained on LD5053 compared to when they were maintained on TL2019S **(B)**. ***, P<0.001, paired t-test, N =7/group. Bodyweights were not significantly different in females maintained on LD5001 **(C)** or LD5053 **(D)** when compared to mice maintained on TL2019S. N =8/group.
