# Supplementary material for: Standard rodent diets differentially impact alcohol consumption, preference, and gut microbiome diversity
Source: Front Neurosci. 2024 May 13;18:1383181. doi: 10.3389/fnins.2024.1383181 (PMC11129685; doi:10.3389/fnins.2024.1383181)
Supplement: Supplementary file 2 [file Data_Sheet_1.PDF]

Table S2A: Water Relative Abundance, P values obtained from TukeyHSD tests on significant genera

Genus	Comparison		
	TL2019S-LD5053	TL2019S-LD5001	LD5053-LD5001
A2	5.00E-02	5.66E-01	5.70E-03
Acetatifactor	1.46E-01	2.47E-02	3.39E-04
Alistipes	4.17E-07	1.14E-06	1.23E-11
Bacteroides	9.84E-01	3.43E-02	4.85E-02
Bifidobacterium	1.60E-02	7.89E-02	7.15E-01
Dubosiella	3.80E-03	3.44E-03	9.99E-01
Escherichia/Shigella	9.97E-01	2.26E-02	2.64E-02
Lachnoclostridium	2.58E-02	6.63E-06	2.76E-03
Lachnospiraceae_NK4A136_group	1.00E+00	4.54E-03	4.61E-03
Lachnospiraceae_UCG-001	1.33E-02	3.66E-03	8.25E-01
Lactobacillus	4.44E-01	6.30E-06	7.70E-05
Roseburia	3.36E-02	1.59E-04	7.30E-07
Ruminococcus	1.55E-03	1.51E-04	5.41E-01
Turicibacter	1.62E-01	2.74E-02	6.28E-01

P value adjusted with Benjamini & Hochberg adjustment

Table S2B: Alcohol Relative Abundance, P values obtained from TukeyHSD tests on significant genera

Genus	Comparison		
	TL2019S-LD5053	TL2019S-LD5001	LD5053-LD5001
Alistipes	2.85E-04	8.25E-01	1.04E-03
Anaeroplasma	9.29E-01	2.51E-03	5.60E-03
Bifidobacterium	1.65E-03	8.96E-05	3.86E-01
Colidextribacter	3.87E-03	1.58E-03	9.13E-01
Dubosiella	5.68E-08	3.14E-08	9.25E-01
Eisenbergiella	7.15E-01	1.49E-02	7.41E-02
Incertae_Sedis	6.49E-01	7.39E-03	4.84E-02
Ruminococcus	1.14E-01	3.61E-05	3.39E-03

P value adjusted with Benjamini & Hochberg adjustment
