# Supplementary figures and images for: Standard rodent diets differentially impact alcohol consumption, preference, and gut microbiome diversity
Source: Front Neurosci. 2024 May 13;18:1383181. doi: 10.3389/fnins.2024.1383181 (PMC11129685; doi:10.3389/fnins.2024.1383181)

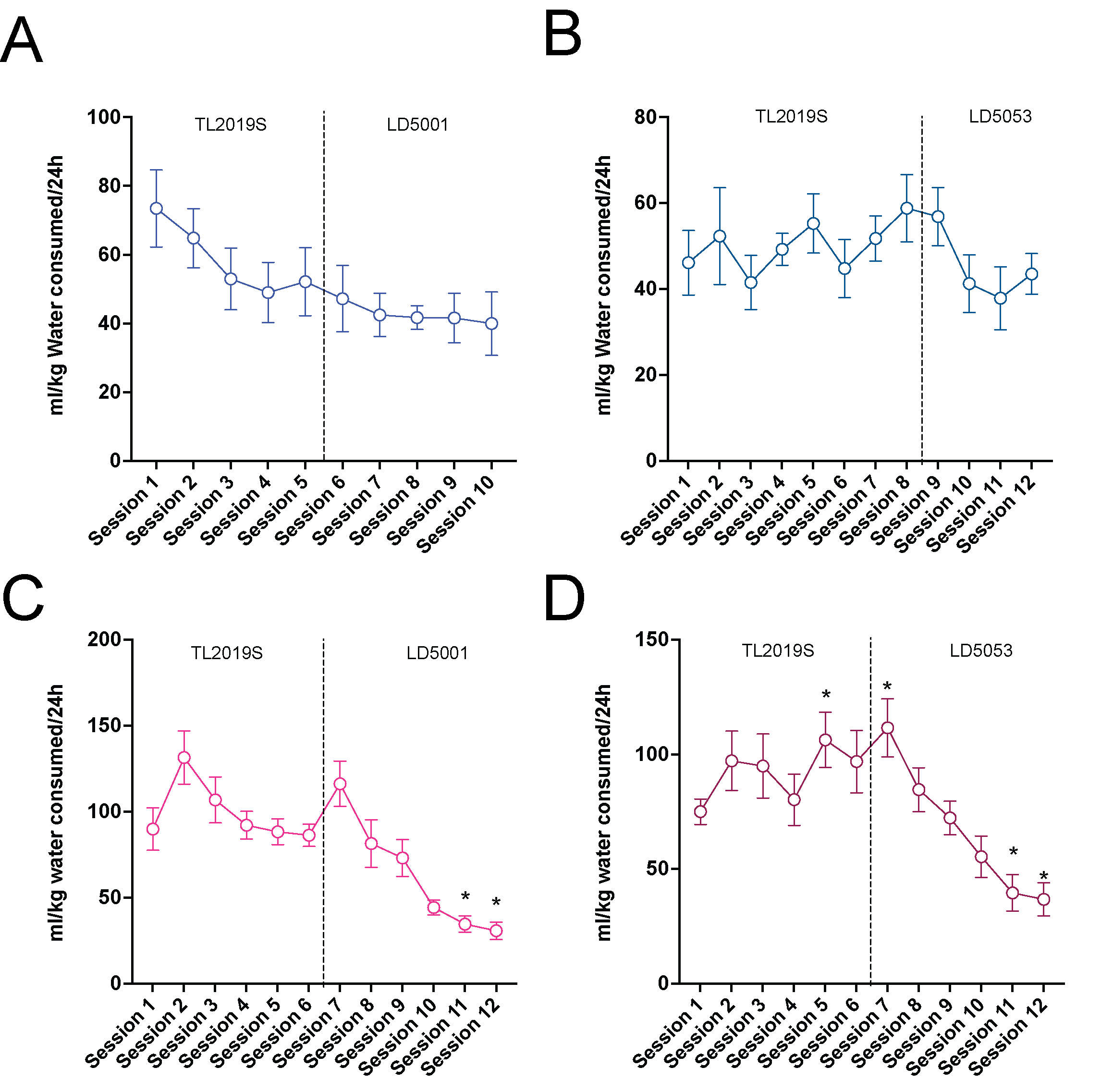

Supplement: Supplementary file 3 [file Image_1.TIF]

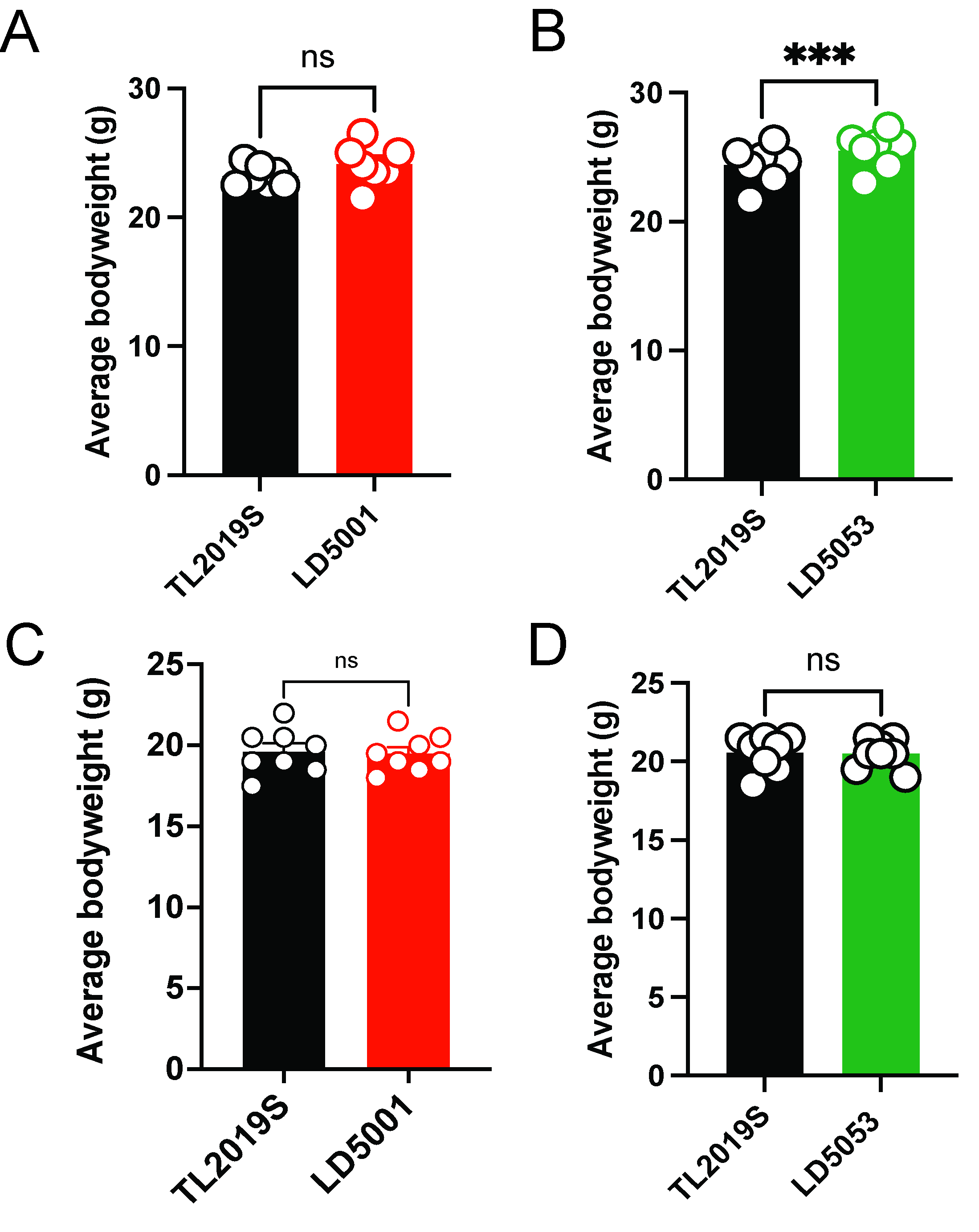

Supplement: Supplementary file 4 [file Image_2.TIF]

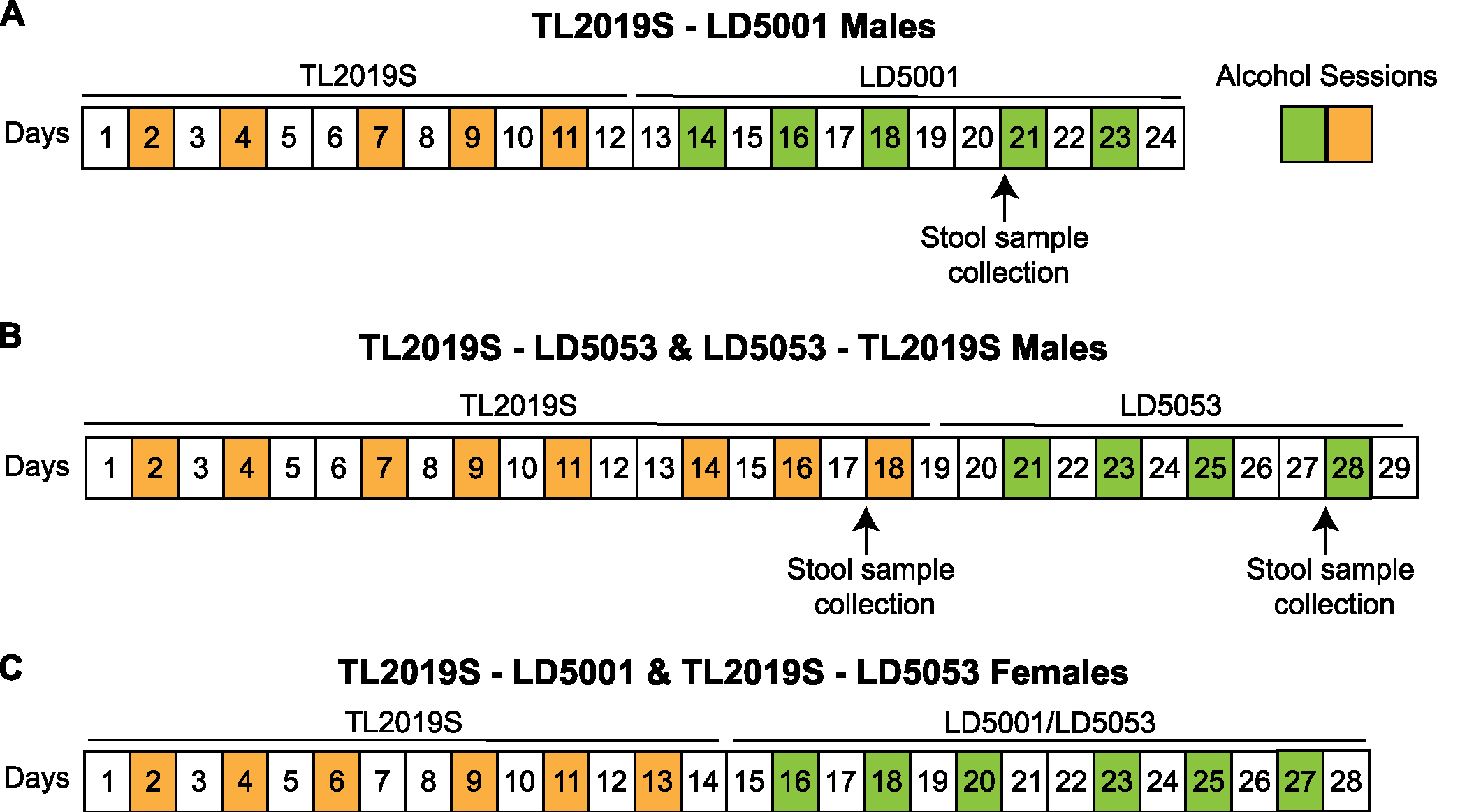

Supplement: Supplementary file 5 [file Image_3.TIF]
